# Average arterial input function for quantitative dynamic contrast enhanced magnetic resonance imaging of neck nodal metastases

**DOI:** 10.1186/1756-6649-9-4

**Published:** 2009-04-07

**Authors:** Amita Shukla-Dave, Nancy Lee, Hilda Stambuk, Ya Wang, Wei Huang, Howard T Thaler, Snehal G Patel, Jatin P Shah, Jason A Koutcher

**Affiliations:** 1 Department of Medical Physics, Memorial Sloan-Kettering Cancer Center, New York, NY, USA; 2 Department of Radiology, Memorial Sloan-Kettering Cancer Center, New York, NY, USA; 3 Department of Radiation Oncology, Memorial Sloan-Kettering Cancer Center, New York, NY, USA; 4 Department of Epidemiology-Biostatistics, Memorial Sloan-Kettering Cancer Center, New York, NY, USA; 5 Department of Surgery, Memorial Sloan-Kettering Cancer Center, New York, NY, USA; 6 Department of Medicine, Memorial Sloan-Kettering Cancer Center, New York, NY, USA

## Abstract

**Background:**

The present study determines the feasibility of generating an average arterial input function (Avg-AIF) from a limited population of patients with neck nodal metastases to be used for pharmacokinetic modeling of dynamic contrast-enhanced MRI (DCE-MRI) data in clinical trials of larger populations.

**Methods:**

Twenty patients (mean age 50 years [range 27–77 years]) with neck nodal metastases underwent pretreatment DCE-MRI studies with a temporal resolution of 3.75 to 7.5 sec on a 1.5T clinical MRI scanner. Eleven individual AIFs (Ind-AIFs) met the criteria of expected enhancement pattern and were used to generate Avg-AIF. Tofts model was used to calculate pharmacokinetic DCE-MRI parameters. Bland-Altman plots and paired Student t-tests were used to describe significant differences between the pharmacokinetic parameters obtained from individual and average AIFs.

**Results:**

Ind-AIFs obtained from eleven patients were used to calculate the Avg-AIF. No overall significant difference (bias) was observed for the transfer constant (K^trans^) measured with Ind-AIFs compared to Avg-AIF (p = 0.20 for region-of-interest (ROI) analysis and p = 0.18 for histogram median analysis). Similarly, no overall significant difference was observed for interstitial fluid space volume fraction (v_e_) measured with Ind-AIFs compared to Avg-AIF (p = 0.48 for ROI analysis and p = 0.93 for histogram median analysis). However, the Bland-Altman plot suggests that as K^trans ^increases, the Ind-AIF estimates tend to become proportionally higher than the Avg-AIF estimates.

**Conclusion:**

We found no statistically significant overall bias in K^trans ^or v_e _estimates derived from Avg-AIF, generated from a limited population, as compared with Ind-AIFs.

However, further study is needed to determine whether calibration is needed across the range of K^trans^. The Avg-AIF obtained from a limited population may be used for pharmacokinetic modeling of DCE-MRI data in larger population studies with neck nodal metastases. Further validation of the Avg-AIF approach with a larger population and in multiple regions is desirable.

## Background

A broad range of tumors [1-3] have been studied clinically by dynamic contrast-enhanced magnetic resonance imaging (DCE-MRI) which monitors the passage of intravenously administered Gadolinium (Gd) contrast agent through tumor tissue. The rate at which the agent passes from the intravascular space to the interstitial space depends on several factors including tumor perfusion, vascular density, and vascular permeability. MRI signal changes depend on these factors and also the leakage (interstitial) space. Tumors are considered to be leaky and to have high perfusion. Three different approaches have been reported for the analysis of the DCE-MRI time course data: a qualitative approach, or subjective assessment of the curve shape; semi-quantitative analysis of the signal time course, such as uptake slope and maximum signal intensity change; and quantitative analysis involving analytical pharmacokinetic modelling of the time course data. The qualitative and semi-quantitative approaches cannot be directly related to physiological parameters, although they do provide clinically useful data. The quantitative approach has proven to be the most sophisticated and provides physiological data [4-6]. Rijpkema et al [4] performed quantitative analyses of DCE-MRI data in eleven patients with tumors, of which six patients had tumor in the head and neck region. Their initial analysis presented the redistribution rate constant k_ep _(= K^trans^/v_e_) maps while the K^trans ^(transfer constant) and v_e _(interstitial fluid space volume fraction) maps could not be expressed in absolute quantitation values due to the scaling factor and were not shown. Kim et al [5] measured the AIF in nine individuals and found the effects of transcytolemmal water exchange to be an important factor in DCE-MRI data analysis. Use of standardized quantitative methods for analysis has been highly recommended so that DCE-MRI data may be compared from different sites, platforms and field strengths and be of widespread use, for example in clinical trials assessing tumor response to therapy [7].

Quantitative analysis of the DCE-MRI data requires knowledge of the arterial input function (AIF): the time-dependent contrast agent concentration in the arterial blood feeding the tissue of interest. The absolute accuracy of the pharmacokinetic parameters K^trans ^and v_e _depends on the AIF accuracy [8]. The K^trans ^and v_e _parameters are clinically relevant and have been used in oncological imaging for tumor detection and to evaluate response to therapy [3,7]. DCE-MRI has become a useful tool also in evaluating head and neck cancers, differentiating tumor from non-tumor in the cervical lymph nodes or lymphoma from squamous cell carcinoma, assessing mandibular invasion, and predicting response to therapy [3,4,6,9-13]. The need for reliable measurements of pharmacokinetic parameters has been the impetus for accurate measurement of AIFs in the carotid artery in head and neck cancer patients [4,5]. However, obtaining individual AIFs for each DCE-MRI study may not be possible in all patients due to data acquisition constraints. This challenge can be overcome if a population-averaged AIF is used [14]. Recent studies in cancer [14,15] have indicated that the use of a high-temporal-resolution population-based AIF allowed assessment of detailed physiological information with a good degree of precision even when individual AIF measurement was not possible. However, the use of Avg-AIF for quantitative analysis of DCE-MRI data in neck nodal metastases has not been tested so far. In the present study, we determined the feasibility of building an average AIF obtained from a limited population of head and neck cancer patients with neck nodal metastases for pharmacokinetic modeling of DCE-MRI data in studies of larger populations, e.g. in clinical trials to examine the effects of treatment on DCE-MRI parameters.

## Methods

### Patients

DCE-MRI was performed in twenty head and neck cancer patients (mean patient age 50 years [range 27–77 years]; 17 males, 3 females, with nodal disease [size >1 cc]; 2 patients stage III and 18 stage IV) before chemo-radiation therapy or surgery. The institutional review board granted exempt status for this retrospective study with a waiver of informed consent for two patients who underwent DCE-MRI between April and September 2005. From February, 2006 to October, 2007 DCE-MRI was performed as part of an ongoing National Institutes of Health (NIH) study investigating the use of MR imaging in patients with head and neck cancers; 18 patients gave informed consent for their participation in the institutional review board-approved NIH study. The study was also compliant with the Health Insurance Portability and Accountability Act. Although 20 patients were recruited, only 11 had AIFs that were included for averaging to produce a population AIF.

### MRI Data acquisition

MRI data were acquired on a 1.5 Tesla GE Excite scanner (General Electric, Milwaukee, WI) with a 4 channel neurovascular phased-array coil (MRI Devices Corporation, Gainesville, FL) for signal reception and a body coil for transmission. The study consisted of MR imaging covering the entire neck. MRI data was acquired with spatial saturation to minimize the flow effects on the MR measurement in the arterial blood vessels. MR acquisition parameters for the neck survey were as follows: rapid scout images, multiplanar (axial, coronal and sagittal) T2-weighted, fat-suppressed, fast-spin echo images (TR = 5000 ms, TE = 102 ms, averages = 2, slice thickness = 5.0 mm and gap = 2.5 mm), multi-planar T1-weighted images (TR = 675 ms, TE = 8 ms, averages = 2, slice thickness = 5.0 mm and gap = 2.5 mm). Standard T1- and T2-weighted imaging were followed by proton density MRI acquired (for the purpose of determining the longitudinal relaxation rate constant R_1 _for each DCE-MRI data point) in the axial plane (TR = 350 ms, TE = 2 ms with a 30° flip angle (α), 2 excitation, 15.63 kHz receive bandwidth, field of view [FOV], slice location and thickness same as in the DCE-MRI scan [see below] and a 256 × 128 matrix), followed by an axial T1-weighted DCE-MRI scan and then post-contrast T1-weighted images in axial and coronal planes. At the beginning of the sixth image set (data point) of the DCE-MRI scan, a bolus of 0.1 mmol/kg GdDTPA contrast agent (Magnevist; Berlex Laboratories, Wayne, NJ, USA) was delivered through an antecubital vein catheter at 2 cc/sec, followed by a saline flush using an MR-compatible programmable power injector (Spectris; Medrad, Indianola, PA, USA). DCE-MRI data were acquired using a 2D-fast multi-phase spoiled gradient echo (FMSPGR) sequence. The entire node was covered contiguously with 5–7-mm thick slices, zero gap yielding 3–6 slices depending on the size of the node with 3.75–7.5 sec temporal resolution. Acquisition parameters included 9 ms TR, 2 ms TE, 30° flip angle (α), 15.63 kHz receive bandwidth, 18–20-cm field of view (FOV), 40–80 time course data points, and a 256 × 128 matrix sized zero filled to 256 × 256 during image reconstruction.

### DCE-MRI Data Analysis

**Figure 1:**
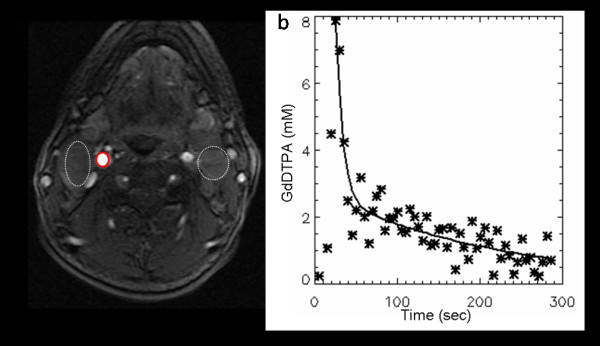
**(a) A post-contrast axial MR image from a head and neck cancer patient extracted from the DCE-MRI scan showing bilateral nodal metastases (dashed white lines) in the neck and the right carotid artery (solid red line)**. (b) An arterial input function (AIF) plot (plasma Gd-DTPA concentration time course). The data points were measured from the region of interest placed within the right carotid artery. The AIF wash-out phase was fitted with a bi-exponential decay function.

Data were exported to a Windows PC and the pharmacokinetic analysis based on the Toft's model was done using in-house software written to display and analyze data using IDL windows version 6.0 (Research Systems, Boulder, CO, USA) [16]. To perform the above pharmacokinetic analysis using the Tofts model [16], a reliable AIF (C_p_(t)) is required. In all patients studied, the carotid artery was visible in most of the image slices. To minimize partial volume effects the image slice that contained the central portion of the artery was used. A region of interest (ROI) was placed within the carotid artery (Figure 1a) and this allowed the direct measurement of the arterial input function through monitoring of the changes in signal intensity (and converting the data into concentration) (Figure 1b). The signal intensity (S) of spoiled gradient echo with contrast agent bolus injection is modeled according to Equation 1, assuming TE << T_2 _so that the final exponential term can be taken as 1, [17]:

(1)

where α is the flip angle, TR is the repetition time, S_0 _is a constant proportional to the proton density of the sample. Based on the method described by Parker et al [18], a calibration curve was generated of signal intensity ratio of T_1_-weighted image to proton density image as a function of T_1_. The details of the phantom experiment are reported by Wang et al [15]. The artery ROI values R_1 _for the DCE image series were obtained from the calibration curve and subsequently converted to Gd-DTPA concentrations. The apparent longitudinal relaxation rate constant, R_1 _(= 1/T_1_), is proportional to C_p _(t) and is given by:

(2)

Where C_p_(t) is the arterial plasma GdDTPA concentration at time t, r_1 _is the contrast agent relaxivity; taken to be 4.1 sec^-1^(mmol/L)^-1 ^at 1.5T [19], and R_10 _is the pre-contrast R_1_. The derived C_p_(t) time course was fitted with a bi-exponential decay function in the wash-out phase [20] to generate the AIF. The Ind-AIF's were inspected to determine whether they followed the "expected" contrast enhancement pattern: enhancement during the early phase followed by signal intensity attenuation. The residual (Sum of residual) and mean square error (MSE) were used as a reference as well as visual inspection done by a physicist. Ind-AIFs were excluded that clearly showed large deviations from the above enhancement pattern shown in Figure 1b (Sum of residual = 0.073; MSE = 0.325). By averaging the eleven individual AIFs (Ind-AIFs) obtained from the carotid artery that satisfied the above criteria, with peak height aligned, an average AIF (Avg-AIF) was obtained. The average AIF was calculated using the equation [16]:

(3)

Where D is the dose (mmole/kg), and amplitudes "a" are normalized for unit dose (so that C_p _is then known for any size dose). For the parameters a_1_, a_2_, m_1 _and m_2 _Mean values are reported as Mean ± SE of the Mean in Table 1. The SE variation show that the changes are large during the initial spike (a) and then reduce to a small variation during the wash out phase (m). This is consistent with previous studies [14,15].

**Table 1:** Parameter values for the average AIF build from the eleven individual AIF's

**Parameters**	**a_1 _(kg/liter)**	**a_2 _(kg/liter)**	**m_1 _(min^-1^)**	**m_2 _(min^-1^)**
Mean	204.07	58.02	16.18	1.31
SE	61.53	17.49	4.87	0.39

Quantitative DCE-MRI analyses of the tumor tissue time course data was done in all the patients for the ROI as well as each pixel within the ROI using histogram analyses [15]. The latter calculates the pixel K^trans ^and v_e _and the median values of these parameters.

For the tumor tissue time course data, ROI were manually drawn by an experienced neuro-radiologist outlining the contrast-enhanced tumor and were used for signal intensity measurements. All the slices containing tumor were outlined and analyzed. The model fitted the tissue contrast agent concentration, C_t_(t), time course for the extraction of the K^trans ^and v_e _parameters from the whole tumor (multiple slices), as shown in the following Kety-Schmidt equation:

(4)

The term that includes plasma volume fraction (v_p_), v_p_C_p_(t), was ignored on the right side of the equation. As shown by Li et al [21] there is sufficient contrast agent extravasation from plasma to interstitium tumor tissue such that K^trans ^and v_e_parameters are adequate for analyses. The exclusion of v_p _may lead to errors in situations when there is less contrast extravasation (such as K^trans ^< 0.001 min^-1^). For each DCE-MRI data set, only the bi-exponential function-fitted AIF wash-out phase was used for C_t_(t) curve fitting with time zero in Eq. [4] set as the time of AIF peak amplitude. This is due to the fact that the arrival of the bolus precedes the apparent rise of tumor tissue signal intensity, defined as signal intensity rising more than one standard deviation (SD) of the signal intensities of the five pre-contrast injection baseline data points.

A paired Student t test was used to evaluate differences in pharmacokinetic parameters resulting from the use of Ind- and Avg-AIFs. Bland-Altman plots [22] were used to explore possible trends across the range of observed ROI means for each parameter. An example of a single slice was plotted to show the pixel-by-pixel variation between the two estimates within the slice.

## Results

**Figure 2:**
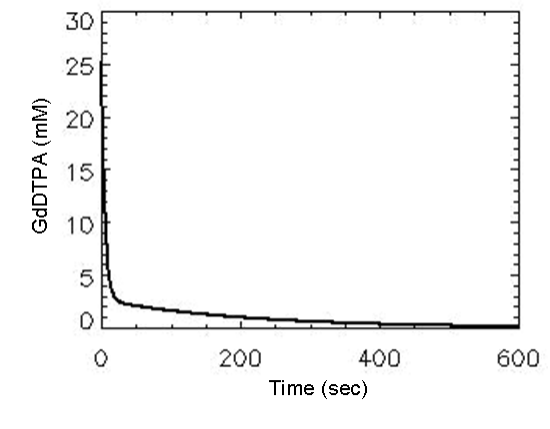
**Average AIF obtained from eleven individually sampled AIFs**.

**Figure 3:**
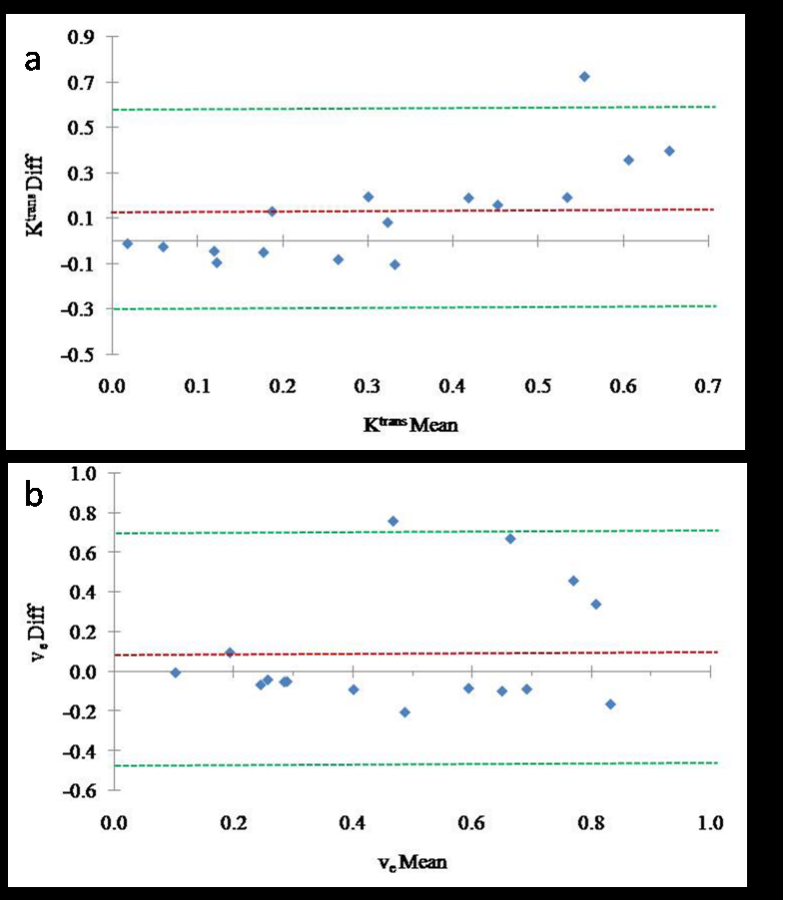
**Bland-Altman plots showing variability of the differences between parameter estimates obtained by the two methods**. Horizontal axes are means and vertical axes are the differences of the paired estimates. Dotted references lines show bias and limits of agreement (mean of differences +/- 2 SD of differences). (a) K^trans ^Ind-AIF – K^trans^Avg-AIF (0.124 +/- .448); (b) v_e _Ind-AIF – v_e_Avg-AIF (0.088 +/- .600).

**Figure 4:**
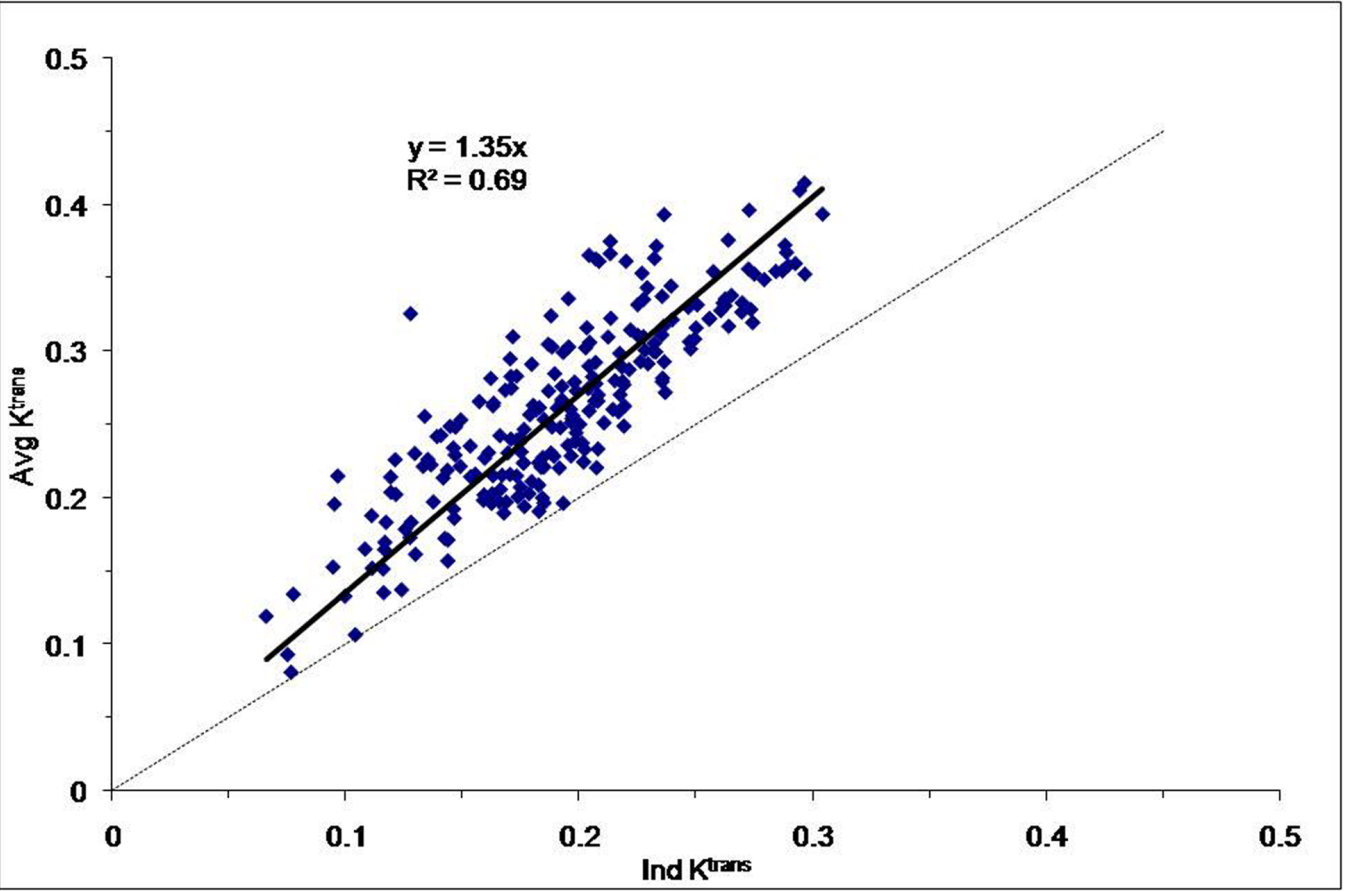
**Scatter plot of pixel K^trans ^values obtained from single-image slice pharmacokinetic modeling analyses of a patient's DCE-MRI data**. The K^trans^extracted with the average AIF (Avg-AIF) approach are plotted against those extracted with the individually measured AIF (Ind-AIF) approach. Both identity line and regression line are shown. The slope of the linear regression is 1.35.

In twenty patients ROI's were drawn in the carotid artery for calculation of the AIF and 55% (eleven patients) had AIF that met the expected pattern of enhancement. The data from these eleven patients was used to build the Average-AIF. The bi-exponential function-fitted Average-AIF with 2 cc/sec contrast agent injection rate is shown in Figure 2. Bland-Altman plots show the values of ROI K^trans ^(Figure 3a) and ROI v_e _(Figure 3b) measured from pharmacokinetic modeling using both Ind-AIFs and the Avg-AIF for the eleven head and neck cancer patient's nodal tumor tissue. For each ROI, the difference between the two estimates is plotted against the mean of the two estimates. Reference lines show the average difference (bias) and limits of agreement (+/- two SD). There were no statistically significant bias between K^trans ^values derived with Ind-AIFs and those derived with the Avg-AIF (Table 2; p = 0.20 for ROI K^trans ^and 0.18 for histogram median K^trans^; paired t test). Similar results were obtained for the v_e _parameter (Table 2). In order to show how Avg-AIF performs on a pixel-by-pixel basis, Figure 4 displays the representative graph for a patient with pixel K^trans ^values within the tumor tissue ROI obtained with the Avg-AIF plotted against those obtained with the Ind-AIF. The plot shows significant linear correlation (p < 0.0001) and a proportional divergence from the identity reference line. The slope value for linear regression through the origin was 1.35. Similar results were obtained from ROIs for the other 10 patients with reliable Ind-AIF measurements. This indicates that the use of Avg-AIF works equally well for both ROI- and pixel-wise analyses.

**Table 2:** DCE-MRI Parameters K^trans^ and v_e _calculated with Ind-AIF and Avg-AIF

**AIF**	Ind-AIF*	Avg-AIF*	**Difference between Ind-AIF and Avg-AIF based parameter estimates**
ROI K^trans ^(min^-1^)	0.35 ± 0.29	0.23 ± 0.12	0.12 ± 0.08^a^
Histogram Median K^trans ^(min^-1^)	0.36 ± 0.29	0.22 ± 0.11	0.14 ± 0.09^b^
ROI v_e_	0.52 ± 0.51	0.43 ± 0.39	0.09 ± 0.06^c^
Histogram Median v_e_	0.37 ± 0.22	0.38 ± 0.19	0.01 ± 0.007^d^

*Mean ± SD; Student's paired t-test for K^trans ^and v_e _values obtained with Ind-AIF and Avg-AIF; Differences between Ind-and Avg-based parameter estimates: ^a^P = 0.20, ^b^P = 0.18, ^c^P = 0.48, ^d^P = 0.93.

## Discussion

DCE-MRI studies have shown great promise in several aspects of head and neck cancer management, including differential diagnosis and assessment of treatment response [3]. To date, the general methods of analysis of the data have ranged from qualitative to quantitative analysis [3-6,9-12]. Quantitative modeling requires accurate AIF measurements [7]. Although it would be ideal to obtain AIFs from individual patients, which is often feasible, in many settings it is not possible to perform an AIF measurement reliably either due to data acquisition constraints or lack of a suitable artery within the imaging FOV from which to obtain an AIF [14,15]. Parker et al suggested that in cases where a reliable AIF was not measured, a high-resolution population-averaged AIF can improve the reproducibility of parameters obtained using kinetic-modelling of DCE-MRI data and that in general only small changes in accuracy can be expected [14]. This study was performed on twenty three patients with cancer in the abdomen [14]. The authors commented that use of the Avg-AIF approach could be a useful alternative to the use of Ind-AIFs, especially for quantitative treatment studies where changes in microvascular properties are more important than the absolute values [14]. Our group has recently published the use of limited population based Avg-AIF for DCE-MRI data analysis in osteosarcomas [15]. In the present study we focus the use of the same principle on a different anatomic site with cancer i.e. head and neck. These preliminary studies can provide basis for large, validation studies and future application in clinical trials which use DCE-MRI parameters as non-invasive MR biomarkers. There is a need for widespread use of quantitative analysis of DCE-MRI data in order to compare and evaluate studies performed at different field strengths which would be independent of instrument platform and acquisition parameters.

Different groups have proposed various methods for analyzing DCE-MRI data for scenarios in which Ind- or Avg-AIFs cannot be obtained easily. These techniques may be an option for analysis of DCE-MRI data after appropriate validation or comparisons with standard methods. Riabkov et al [23] estimated the kinetic parameters without input functions using multichannel blind identification methods and iterative quadratic maximum-likelihood (IQML) gave the most accurate estimates. Yankeelov et al [24] and Walker-Samuel et al [25,26] have proposed a method that compares the tissue of interest (TOI) curve shape to that of a reference region (RR), thereby eliminating the need for direct AIF measurements when a reliable AIF is not obtainable. Yang et al [27] proposed the double-reference-tissue method, which assumes that the AIFs of the two reference tissues have the same shape. The elegant simulations used in their study need to be tested in more complicated tissues, such as tumor tissue.

Clinical requirements for diagnosis often dictate large imaging spatial coverage and high image resolution, which result in poor temporal resolution for DCE-MRI acquisition. Roberts et al [28] showed that AIFs sampled at low temporal resolution introduced an unpredictable degree of error in the quantitative analysis. In such cases the use of Avg-AIF obtained from acquisitions with higher temporal resolution would be the method of choice. In the present study, the Avg-AIF was obtained from DCE-MR images of sufficient temporal resolution, and therefore it may be used to analyze DCE-MRI data that were acquired with poorer temporal resolution but otherwise with the same contrast injection set up, including dose, injection site and injection rate. In the present study ROIs were drawn on all arteries visible on the MR images but reliable AIF measurement was obtained only from the section that contained the central portion of the artery. This was because other sections showed small vessels containing only a small number of pixels, leading to a partial volume effect, or because other sections showed the bifurcation of the common carotid artery. Care was taken by the physicist during the selection process of Ind-AIFs so as to not pre-determine the outcome of the Avg-AIF, but rather to remove patient data that largely deviated from the expected contrast enhancement pattern.

A bi-exponential function was used for data analysis. Consistent use of this function for AIF curve fitting will not introduce systematic errors in longitudinal comparisons of changes in pharmacokinetic parameters caused by treatment. The use of an Avg-AIF implies that resulting parameter estimates will be higher than Ind-AIF estimates for some cases and lower for others. However, there is added benefit in the implicit gain in precision of the pooled or Avg-AIF and the possibility of parameter estimation when Ind-AIFs are not available. Although the sample size was small, other studies of cancers such as sarcoma have reported similar findings for Avg-AIF from small population [15]. Further validation of the Avg-AIF approach with a larger population and in multiple regions is desirable.

## Conclusion

In conclusion, this study shows that parameter estimates derived from an Avg-AIF, generated from a limited population, yield similar results to estimates derived from Ind-AIFs. The Avg-AIF may be useful for pharmacokinetic modeling of DCE-MRI data in studies of larger populations of patients with neck nodal metastases where precise Ind-AIFs may not be available for all cases, e.g., in clinical trials to examine the effects of drugs on DCE-MRI parameters.

## Abbreviations

DCE-MRI: Dynamic contrast-enhanced MRI; AIF: arterial input function; K^trans^: transfer constant; v_e_: interstitial fluid space volume fraction.

## Competing interests

The authors declare that they have no competing interests.

## Authors' contributions

1) All authors (ASD, NL, HS, YW, WH, SGP, JPS and JAK) have made contributions to conceptions and design of this study. NL, SGP, JPS referred patients for the study. ASD, YW, HS, WH, JAK made contribution in acquisition of data, and interpretation of data; 2) ASD, WH and JAK have been involved in drafting the manuscript or revising it critically for important intellectual content; and 3) All authors have given final approval of the version to be submitted to BMC Medical Physics.

## Pre-publication history

The pre-publication history for this paper can be accessed here:


